# ELAVL1 promotes ferroptosis via the TRIM21/HOXD8 axis to inhibit osteogenic differentiation in congenital pseudoarticular tibia‐derived mesenchymal stem cells

**DOI:** 10.1002/ccs3.70016

**Published:** 2025-05-21

**Authors:** Weihua Ye, Zheng Liu, Yaoxi Liu, Han Xiao, Qian Tan, An Yan, Guanghui Zhu

**Affiliations:** ^1^ Orthopedic Department The Affiliated Children’s Hospital of Xiangya School of Medicine, Central South University (Hunan Children’s Hospital), Hunan Provincial Key Laboratory of Pediatric Orthopedics Changsha Hunan China

**Keywords:** congenital tibial pseudoarthrosis, ELAVL1, ferroptosis, osteogenic differentiation, TRIM21

## Abstract

Osteogenic differentiation of mesenchymal stem cells (MSCs) was strongly correlated with the progression of congenital tibial pseudoarthrosis (CPT). Activation of ferroptosis inhibited osteogenic differentiation of MSCs. ELAV‐like RNA binding protein 1 (ELAVL1) is a key factor in promoting ferroptosis. This study aimed to elucidate the mechanism of ELAVL1 in the osteogenic differentiation of CPT periosteum‐derived MSCs. Osteogenic differentiation of CPT periosteum‐derived MSCs was detected by ARS and ALP staining. Fe^2+^ content and lipid reactive oxygen species content were measured using commercial kits. Molecular interactions were verified using RIP, RNA pulldown, and Co‐IP. The ubiquitination level of homeobox gene D8 (HOXD8) was detected using Co‐IP. Expression of ELAVL1 and tripartite motif containing 21 (TRIM21) was upregulated in CPT periosteum‐derived MSCs, whereas HOXD8 expression was downregulated. Moreover, knockdown of ELAVL1 or TRIM21 inhibited ferroptosis and promoted osteogenic differentiation of CPT MSCs. TRIM21 overexpression reversed the effect caused by knockdown of ELAVL1. Mechanistically, ELAVL1 upregulated TRIM21 by increasing the stability of TRIM21, which ubiquitinated and degraded HOXD8. ELAVL1 bound to TRIM21, which promoted ubiquitination and degradation of HOXD8, thereby promoting ferroptosis to inhibit osteogenic differentiation of CPT MSCs.

## Graphical abstract

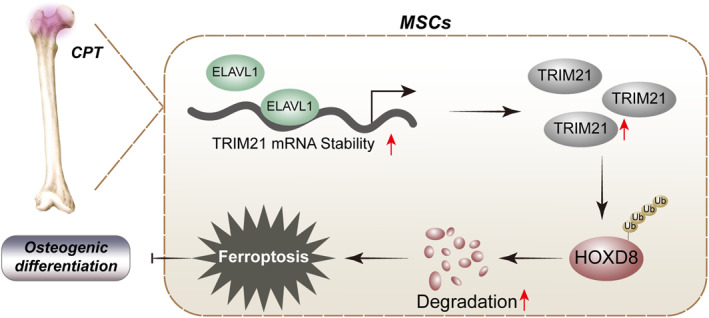

## 1 INTRODUCTION

Congenital pseudoarthrosis of the tibia (CPT) is rare disorders in pediatric orthopedics, with an incidence of approximately 1/140000–1/250000.^1^ Currently, the treatment of CPT is mainly surgical, but its outcome is not satisfactory.^2^ Therefore, it is critical to seek treatment with CPT. According to the report, CPT was caused by periosteum lesions.^3^ In addition, recent studies demonstrated that inhibition of osteogenic differentiation of periosteum‐derived mesenchymal stem cells (MSCs) promoted the progression of CPT.^4^ Our previous study also found that the osteogenic differentiation capacity of CPT MSCs was significantly lower than that of normal MSCs.^5^ Therefore, enhancing osteogenic differentiation of MSCs may be a potential approach for future treatment of CPT. Ferroptosis is a nonapoptotic form of cell death mode that is dependent on iron and reactive oxygen species (ROS) accumulation.^6^ Recent studies suggested that accumulation of excess iron led to ferroptosis in MSCs.^7^ In addition, stimulating the ferroptosis pathway suppressed the ability of MSCs to differentiate into osteoblasts, thereby contributing to osteoporosis.^8^ However, whether ferroptosis affects CPT has not been reported.

The homeobox gene D8 (HOXD8) belongs to the HOX gene family, which demonstrated that HOXD8 promoted osteogenic differentiation of MSCs. For example, lncRNA AIS promoted RUNX2 transcription by upregulating HOXD8 expression, which ultimately promoted osteogenic differentiation of bone marrow MSCs.^9^ Previous research by the authors found thatmiR‐30a inhibited osteogenic differentiation of CPT MSCs through suppressing HOXD8 expression.^5^ In addition, another study of ours also demonstrated that methyltransferase‐like three enhanced osteogenic differentiation of CPT MSCs via increasing HOXD8 expression.^10^ However, the role of HOXD8 in ferroptosis is unclear. In addition, studies have reported that HOX family genes have an inhibitory effect toward ferroptosis, such as HOXD10 and HOXA11‐AS.^11^, ^12^ Therefore, we hypothesized that HOXD8 may also have an inhibitory effect on ferroptosis. The etiology of CPT would likely to promote ferroptosis by inhibiting HOXD8 expression, which in turn suppresses osteogenic differentiation of CPT MSCs.

The ubiquitin‐proteasome system serves as the primary protein degradation pathway in eukaryotic organisms.^13^ E3 ubiquitin ligases, with their strict substrate recognition and ubiquitin chain generation specificity, play the most critical role in ubiquitination.^14^ Tripartite motif containing 21 (TRIM21), a member of the TRIM protein family, which functions as an E3 ubiquitin ligase activity, responsible for protein degradation.^15^ For example, TRIM21 inhibited DNA replication stress‐induced activation of CHK1 by ubiquitinating CLASPIN, which ultimately promoted tumorigenesis.^16^ Notably, Xian et al.^15^ reported that TRIM21 prevented osteogenic differentiation of MSCs by enhancing the degradation of Akt. Interestingly, we found that TRIM21 might interact with HOXD8 by ubiBrowser (http://ubibrowser.ncpsb.org.cn) prediction. Additionally, TRIM21 also promoted ferroptosis in acute kidney injury.^17^ Therefore, we initially hypothesized that TRIM21 may ubiquitinate HOXD8 and promotes ferroptosis, thereby enhancing osteogenic differentiation of MSCs in CPT.

RNA‐binding proteins (RBPs) are key regulators of gene expression, affecting the localization, translation, and stability of target genes.^18^ ELAV‐like RNA binding protein 1 (ELAVL1/HuR) is one of the most common RNA‐binding proteins that regulates RNA stability in multiple tissues.^19^ Numerous evidences suggested that ELAVL1 promoted ferroptosis in a variety of cells by regulating mRNA stability.^20^, ^21^ For example, ELAVL1 facilitated ferroptosis in hepatic stellate cells by enhancing BECN1 mRNA stabilization.^21^ A study revealed that knockdown of ELAVL1 could alleviate diabetic osteoporosis by inhibiting ferroptosis and promoting osteoblast differentiation.^22^ Another study showed that ELAVL1 inhibited osteogenic differentiation of bone marrow‐derived MSCs by suppressing the expression of multiple osteogenic genes.^19^ However, the influence of ELAVL1 on osteogenic differentiation of CPT MSCs is unknown. Interestingly, we discovered that ELAVL1 might bind to TRIM21 mRNA by starBase (https://starbase.sysu.edu.cn/index.php) prediction. Therefore, ELAVL1 may inhibit osteogenic differentiation of periosteum‐derived MSCs by increasing the stability of TRIM21 mRNA to promote ferroptosis in CPT.

This study elucidated that ELAVL1 promoted ferroptosis by increasing the stability of TRIM21 mRNA and facilitating the ubiquitination of HOXD8, ultimately inhibiting osteogenic differentiation of CPT MSCs. This research sought to offer a novel theoretical foundation to better understand the pathological mechanisms of CPT and new approaches for its clinical management.

## 2 MATERIALS AND METHODS

### 2.1 Cell culture

MSCs were isolated from periosteum of patients with CPT (*n* = 3, CPT MSCs) or healthy iliac periosteum of patients with hip dysplasia (*n* = 3, normal MSCs) attending the Department of Orthopedics, Hunan Children's Hospital. Detailed isolation and culture steps were as described in our previous study.^5^, ^10^ This study involved the use of signed informed consent forms for all participants, and the work was overseen by the Ethics Committee of Hunan Children's Hospital.

293 T cells were purchased from ATCC (Manassas, VA, USA), was maintained in a controlled culture environment, utilizing DMEM medium (Invitrogen, CA, USA) supplemented with 10% fetal bovine serum at 37°C with 5% CO_2_ conditions. Cells in logarithmic phase were taken for subsequent experiments.

### 2.2 MSCs' osteogenic differentiation

Selection of CPT MSCs for 2–3 generations. After washing with PBS, CPT MSCs were cultured in HUXMX‐90021 medium (Cyagen Biotech, Suzhou, China) for 21 days. On the other hand, Normal MSCs were still cultured using *α*‐minimum essential medium. In the 14th day of induction, early osteogenic differentiation of MSCs were detected using the alkaline phosphatase (ALP) assay kit (P0321S, Beyotime, Shanghai, China). After 21 days, osteogenic differentiation was assayed with the Alizarin Red S (ARS) kit (C0148S, Beyotime).

### 2.3 Cell transfection and infection

For TRIM21 and ELAVL1 knockdown, shRNA oligos were bonded to pSIREN‐RetroQ (Clontech, CA, USA) with TRIM21 or ELAVL1 overhangs for the construction lenti‐pSIREN‐RetroQ‐sh‐TRIM21 orlenti‐pSIREN‐RetroQ‐sh‐ELAVL1. Its negative control was lenti‐pSIREN‐RetroQ‐sh‐NC. TRIM21 or HOXD8 was overexpressed by cloning its coding sequence into the lentiviral plasmid PLVX (Hanbio Biotech, Shanghai, China). The constructed lentiviral vector and its helper packaging original vector plasmid were cotransfected into 293 T cells using the lentiviral packaging kit (LPK001, Genchem, Shanghai). After 48 h of transfection, the cell culture was collected and then concentrated and purified. After calibrating the viral titer, CPT MSCs (5 × 10^4^ cells) were infected with the viral solution (2.5 × 10^8^ TU/mL). CPT MSCs were then screened for successful lentivirus infection with 2 μg/mL puromycin (InvivoGen, CA, USA).

The full‐length cDNA sequence of TRIM21 or HOXD8 was cloned into the pCMV‐Flag vector (Beyotime Biotech) or pCMV‐Myc‐vector (Snapgene, California, USA), respectively. The pCMV‐HA‐Ub plasmid was procured from GenePharma (Shanghai, China). The pCMV‐Flag‐TRIM21 plasmid and pCMV‐Myc‐HOXD8 or pCMV‐Flag‐TRIM21, pCMV‐Myc‐HOXD8, and pCMV‐HA‐Ub plasmids were transfected into 293 T cells by the Lipofectamine 2000 (Invitrogen) for 48 h. Subsequently, cells were collected for subsequent experiments.

### 2.4 Cell treatment

To verify the activation status of ELAVL1, CPT MSCs were treated with the PI3K inhibitor LY294002 (HY‐10108, MedChemExpress, USA) or the NF‐κB inhibitor BAY 11‐7082 (HY‐13453, MedChemExpress, USA). After 24 h, the cells were collected to detect the expression of relevant molecules.

### 2.5 Western blot

After extraction of total proteins from the cells, protein concentration was determined with the BCA protein concentration assay kit (Beyotime). SDS‐PAGE was used to separate proteins and then electrotransferred onto PVDF membranes (Roche, Basel, Switzerland). The membranes were incubated with 5% skimmed milk for 1.5 h. PVDF membranes were subsequently exposed to anti‐TRIM21 (1:1000, ab91423, Abcam, MA, USA) antibody, anti‐HOXD8 (1:1500, ab228450, Abcam) antibody, anti‐ELAVL1 (1:2000, ab200342, Abcam) antibody, anti‐Runt‐related transcription factor 2 (RUNX2, 1:1000, ab236639, Abcam) antibody, antiosteopontin (OPN, 1:1500, ab214050, Abcam) antibody, antiosteocalcin (OCN, 1:1000, ab93876, Abcam) antibody, anti‐GPX4 (1:1000, ab125066, Abcam) antibody, anti‐Solute carrier family 7 member 11 (SLC7A11, 1:2000, #98051, Cell Signaling Technology, MA, USA) antibody, antitotal AKT(1:2000, #9272, Cell Signaling Technology) antibody, anti‐p‐AKT (1:2000, #4060, Cell Signaling Technology) antibody, anti‐NF‐κB p65 (1:1000, #8242, Cell Signaling Technology) antibody, anti‐U1 SnRNP70 (1:1000, ab316762, Abcam) antibody, and anti‐GAPDH (1:5000, ab9485, Abcam) antibody overnight. The next day, the membranes were treated with goat antirabbit IgG (1:5000, ab6721, Abcam) antibody for 1 h. Subsequently, protein bands were colorized with chemiluminescent substrates (Meron Bio, Dalian, China) and observed on an ImageQuant LAS 4000 imaging system (GE Healthcare, CA, USA). GAPDH was chosen as endogenous normalization control.

### 2.6 RT‐qPCR

Total RNA was isolated from CPT MSCs using TRIzol reagent (Takara, Dalian, China). Reverse transcription was performed by using the PrimeScript™ RT reagent Kit (Takara). Then, the expression of ELAVL1 and TRIM21 were checked with TB Green® Premix Ex Taq™ II (Takara). The mRNA levels of the target genes were determined using the 2^−ΔΔCt^ method, with GAPDH serving as the internal reference. The primers were as follows:

ELAVL1‐F: 5′‐AACTACGTGACCGCGAAGG‐3′
ELAVL1‐R: 5'‐CGCCCAAACCGAGAGAACA‐3′
TRIM21‐F: 5′‐GTCCTGGAAAGGAGTGAGTCC‐3'
TRIM21‐R: 5′‐CTGAAAGTATCAGCCACGGATT‐3′
GAPDH‐F: 5′‐CCAGGTGGTCTCCTCTGA‐3′
GAPDH‐R: 5′‐GCTGTAGCCAAATCGTTGT‐3′

### 2.7 Immunofluorescence (IF) staining

First, CPT MSCs were fixed with 4% paraformaldehyde for 15 min. Then, MSCs were treated with 5% goat serum for 1h. MSCs were then incubated with anti‐TRIM21 (1:500, ab91423, Abcam) and anti‐ELAVL1 (1:250, ab 200342, Abcam) antibodies at 4°C overnight. The MSCs were then cultured with the primary antibody corresponding to the secondary antibody for 1 h. MSCs were then incubated with DAPI for 5 min away from light. After treatment with an antifluorescent bursting agent, three random fields of view of each group were selected and observed under a fluorescence microscope for imaging. The fluorescence intensity was then analyzed using the ImageJ software.

### 2.8 RNA immunoprecipitation (RIP) assay

RIP assay using the Magna RIP Kit (Millipore, MA, USA). MSCs (1 × 10^7^ cells) were exposed to lysis buffer and cell extracts were collected. 5 μg of anti‐ELAVL1 (1:250, ab200342, Abcam) antibody or IgG (1:100, ab205718, Abcam) antibody was conjugated to protein A/G beads for 30 min. Cell samples incubated with IgG are considered as negative control. Subsequently, cell extracts were added to mixture and incubated at 4°C overnight. Then, RNA was extracted using RNAiso Plus (Takara), and TRIM21 mRNA levels were analyzed using the RT‐qPCR.

### 2.9 RNA pulldown

After cell lysis, the cell lysate was collected and centrifuged. The lysate was incubated with the biotin‐labeled TRIM21 RNA probe overnight at 4°C. RNA‐protein complexes were obtained. On the following day, the RNA‐protein complex was mixed with the streptavidin magnetic beads and incubated for 2 h at 4°C to form the biotin RNA probe‐streptavidin magnetic bead complex. After elution, the protein was separated. Subsequently, 2 × SDS PAGE loading buffer (Takara) was supplemented with the separated proteins and denatured at 95°C for 10 min. Finally, ELAVL1 protein expression was detected using the western blot.

### 2.10 ALP staining assay

CPT MSCs were fastened with 4% paraformaldehyde for 30 min. ALP staining was carried out on CPT MSCs according to ALP assay kit instructions (SCR004, Merck, Beijing, China). The stained cells were observed and counted under a microscope (Olympus, Tokyo, Japan).

### 2.11 ARS staining

Matrix mineralization of CPT MSCs was assessed using the ARS kit (C0148S, Beyotime). CPT MSCs were fixed for 30 min with 4% paraformaldehyde. After washing, CPT MSCs were stained using ARS for 30 min. At last, CPT MSCs were washed with distilled water and photographed under the Olympus microscope.

### 2.12 Bioinformatic analysis

Multiple binding sites for ELAVL1 and TRIM21 mRNA were predicted using the starBase (https://starbase.sysu.edu.cn/index.php). The potential ubiquitination modification of HOXD8 by the E3 ubiquitin ligase TRIM21 was predicated by ubiBrowser (http://ubibrowser.ncpsb.org.cn). The potential binding sites between HOXD8 and GPX4 and SLC7A11 promoter were predicted using JASPAR (https://jaspar.elixir.no/). StarBase database (https://starbase.sysu.edu.cn/index.php) was used to predict the binding of ELAVL1 to the 3′ untranslated region (3′ UTR) of TRIM21, and the specified binding site of ELAVL1 (GTTT site) in TRIM21 3′ UTR was predicted using the RBPDB database (http://rbpdb.ccbr.utoronto.ca/advanced_search.php).

### 2.13 Fe^2+^ assay

Upon successful lentiviral infection, MSCs (1 × 10^4^ cells) were treated with erastin (10 μM) for 48 h. Subsequently, experiments were performed following Fe^2+^ content assay kit (BC5415, Solarbio, Beijing, China) instructions. Absorbance was assayed using a Multiskan SkyHigh microplate (Thermo Fisher Scientific). The amount of Fe^2+^ was calculated from the standard curve.

### 2.14 C11‐BODIPY staining for detection of lipid ROS content

CPT MSCs were further treated with 10 μM erastin. CPT MSCs were digested with 0.25% tryptase. After centrifugation, BODIPY 581/591 C11 staining working solution (S0043M, Beyotime) was supplemented with the cells. The cell suspension (1 × 10^7^ cells/well) was incubated at 37°C for 30 min. Subsequently, the cell suspension was centrifuged before discarding the supernatant. After centrifugation, cells were resuspended by PBS and analyzed using the flow cytometry (CytoFLEX, FL, USA). Simultaneously, CPT MSCs were seeded in six‐well plates and coincubated with the BODIPY 581/591 C11 working solution. Subsequently, the lipid peroxidation was then visualized via the LSM710 confocal microscopy (Zeiss, Jena, Germany), where the oxidized and reduced forms of BODIPY C11 581–591 were excited at wavelengths of 488 and 561 nm, respectively. Quantification of lipid peroxidation was based on the increase in green fluorescence emission, observed near 510 nm (with a detection range of 500–550 nm). Lipid ROS levels were analyzed using the Fiji/ImageJ software.

### 2.15 Coimmunoprecipitation (Co‐IP) assays

Flag‐TRIM21 and Myc‐HOXD8 were expressed in 293 T cells. 293 T cells and CPT MSCs were lyzed using RIPA (Meilunbio). Co‐IP experiments were performed with the Pierce^TM^ HA‐Tag Co‐IP Kit (Thermo Fisher Scientific). The cell lysate supernatant was incubated with the appropriate antibody overnight at 4°C. Subsequently, protein A/G beads were supplemented with the above mixture and incubated for 4 h. Finally, the precipitated proteins were eluted and identified using western blot analysis. The relevant antibody information is as follows: anti‐HOXD8‐antibody (1:50, mouse monoclonal, sc‐517116, Santa Cruz Biotechnology, for immunoprecipitation; 1:1000, sc‐517116, Santa Cruz Biotechnology, for immunoblotting); anti‐TRIM21‐antibody (1:1000, rabbit polyclonal, ab91423, Abcam, for immunoblotting); anti‐Flag‐antibody (1:30, ab205606, Abcam); anti‐Myc‐antibody (1:100, ab32072, Abcam); and IgG (1:100, ab205718, Abcam). Cell samples incubated with IgG are considered as negative control.

### 2.16 TRIM21 mRNA stability detection

The treated CPT MSCs were incubated with 5 mg/mL of actinomycin D (Sigma, MO, USA) for 0, 2, and 4 h. The expression of remaining TRIM21 mRNA was detected using RT‐qPCR.

### 2.17 HOXD8 protein stability assay

After infection with lenti‐sh‐TRIM21 or lenti‐sh‐NC, CPT MSCs were treated with CHX (40 μg/mL, Meilunbio) for 0, 15, 30, 60, 120, and 240 min. To inhibit proteasomal degradation, CPT MSCs were treated with 10 μM of MG132 (S1748, Beyotime). Subsequently, HOXD8 protein expression was tested using the western blot.

### 2.18 Ubiquitination and degradation experiments

For the detection of exogenous ubiquitination, Flag‐TRIM21, Myc‐HOXD8, and HA‐Ub were expressed in 293 T cells. Subsequently, Flag‐TRIM21 and Myc‐HOXD8 were incubated with HA‐Ub for 6 h at 37°C and 293 T cells were treated with 5 μmol/L of MG132 (S1748, Beyotime). The reaction was terminated by adding SDS–PAGE loading buffer, followed using the western blot. *β*‐actin was used as an internal reference.

For endogenous ubiquitination detection, 2 μg of lenti‐sh‐TRIM21 were transfected into CPT MSCs and the cells were treated with 5 mol/L MG132 (S1748, Beyotime). The ubiquitination level of HOXD8 was analyzed using the western blot.

### 2.19 Chromatin immunoprecipitation (ChIP) assays

CPT MSCs were treated with formaldehyde to covalently crosslink; then, the crosslinked samples were lyzed and fragmented into smaller pieces by sonication. The fragmented chromatin was incubated with HOXD8 antibody (1:50, mouse monoclonal, sc‐515357, Santa Cruz Biotechnology, verified by immunoblotting) or IgG (1:100, ab205718, Abcam). This antibody‐protein‐DNA complex was then captured using protein A/G beads and then eluted from the beads. Cell samples incubated with IgG are considered as negative control. Finally, the released DNA was purified and analyzed using quantitative PCR to measure the enrichment of specific DNA sequences. The primers of related molecules were as follows:

GPX4‐F: 5′‐GCACTTTTGGGAAGCTGAGG‐3′
GPX4‐R: 5′‐AACCTCCCGTCTCTGCTTC‐3′
SLC7A11‐F: 5′‐CTTCTTTGGGAGTTTTAGCAGGA‐3′
SLC7A11‐R: 5′‐TGACACCACCTCAATGCTTTG‐3′

### 2.20 Dual luciferase reporter assays

To investigate the binding between ELAVL1 and TRIM21, the 3′ UTR of TRIM21 containing either the wild‐type or mutant (G‐to‐A mutation) GTTT site (ELAVL1 binding site) was cloned into the GV272 vector (Genechem, Shanghai, China). These constructs were cotransfected into 293 T cells along with lenti‐shNC and lenti‐shELAVL1. After 48 h, the luciferase activity of each group was measured using the Dual Luciferase Reporter Assay Kit (E1910, Promega, USA).

### 2.21 Statistical analysis

Statistical analyses were performed using the SPSS 20.0 software (SPSS, IL, USA). One‐way ANOVA was used for multiple group comparisons followed using Tukey's test. Quantitative data were presented as the mean ± standard deviation (SD). All experiments were performed in triplicate independently. The threshold for statistical significance was set at *p* < 0.05.

## 3 RESULTS

### 3.1 Knockdown of TRIM21 inhibited ferroptosis to promote osteogenic differentiation of CPT MSCs

**FIGURE 1:**
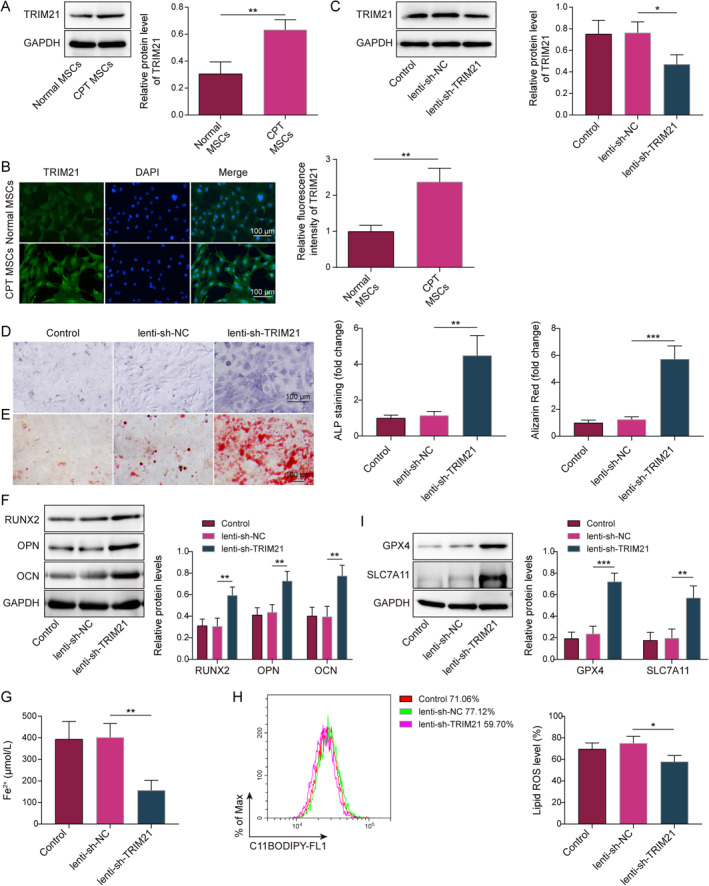
Knockdown of TRIM21 inhibited ferroptosis to promote osteogenic differentiation of CPT mesenchymal stem cells (MSCs) were derived from normal iliac periosteum and CPT patient periosteum, respectively (*n* = 3). (A) Western blot was performed to detect TRIM21 protein expression; (B) expression and distribution of TRIM21 wee analyzed using IF staining (scale bar = 100 μm). CPT MSCs were infected with lenti‐sh‐TRIM21 or lenti‐sh‐NC and induced osteogenic differentiation. (C) TRIM21 expression was tested using the western blot; (D) the treated CPT MSCs were subjected to alkaline phosphatase staining (scale bar = 100 μm); (E) mineralization of the cellular matrix was examined through ARS staining (scale bar = 100 μm); (F) osteogenesis‐related marker genes RUNX2, OPN, and OCN were examined using the western blot. Erastin (10 μM) was added to the above groups of cells to activate ferroptosis in MSCs and (G and H) Fe^2+^ content and lipid reactive oxygen species content in MSCs were determined using the kit; (I) GPX4 and SLC7A11 expression in MSCs were checked using the western blot. *n* = 3. *p < 0.05, **p < 0.01, and ***p < 0.001.

Compared with normal periosteal‐derived MSCs, TRIM21 expression was significantly elevated in CPT MSCs (Figure 1A,B). TRIM21 was present in both the cell nucleus and cytoplasm (Figure 1B). Next, CPT MSCs were infected with lenti‐sh‐TRIM21 and induced osteogenic differentiation. As shown in Figure 1C, TRIM21 was successfully knocked down. Knockdown of TRIM21 enhanced osteogenic differentiation and extracellular matrix mineralization in CPT MSCs (Figure 1D,E). Meanwhile, knockdown of TRIM21 markedly upregulated the expression of RUNX2, OPN, and OCN markers related to osteogenesis in CPT MSCs (Figure 1F). These results suggested that knockdown of TRIM21 promoted osteogenic differentiation of CPT MSCs. Therefore, we further induced ferroptosis and found that knockdown of TRIM21 significantly reduced Fe^2+^ and lipid ROS content in CPT MSCs (Figure 1G,H; Supporting Information S1: Figure S1A). In addition, TRIM21 knockdown increased expression of ferroptosis inhibition‐related factors GPX4 and SLC7A11 in CPT MSCs (Figure 1I). Therefore, TRIM21 was upregulated in the periosteum‐derived MSC, and knockdown of TRIM21 inhibited ferroptosis and promoted osteogenic differentiation of CPT‐MSCs.

### 3.2 TRIM21 ubiquitinated and degraded HOXD8

**FIGURE 2:**
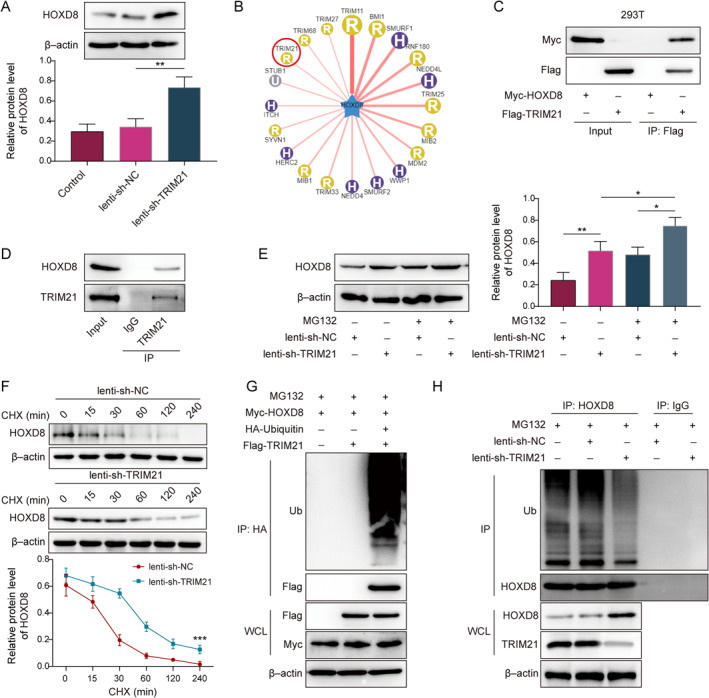
TRIM21 ubiquitinated and degraded HOXD8. (A) TRIM21 was knocked down in CPT mesenchymal stem cells (MSCs), and HOXD8 expression was checked via the western blot. (B) The relationship of TRIM21 binding with HOXD8 was predicted by UbiBrowser. (C) Co‐IP assays were used to test binding of TRIM21 to HOXD8 in 293 T cells overexpressing Flag‐TRIM21 and Myc‐HOXD8. (D) Co‐IP experiments were applied to analyze the binding of TRIM21 to HOXD8 in CPT MSCs. (E) CPT MSCs were treated with MG132 to knockdown TRIM21, and the western blot was used to evaluate HOXD8 expression. (F) The influence of TRIM21 knockdown on HOXD8 stability was assessed via the western blot after CHX treatment of CPT MSCs for 0, 15, 30, 60, 120, and 240 min. (G) In 293 T cells cotransfected with Flag‐TRIM21, Myc‐HOXD8, and HA‐Ub, ubiquitination and degradation of HOXD8 were analyzed through Co‐IP. (H) Co‐IP was employed to measure ubiquitination and degradation of HOXD8 in CPT MSC cells with knockdown of TRIM21. *n* = 3. *p < 0.05, **p < 0.01, and ***p < 0.001.

Initially, we verified the direct regulatory relationship between HOXD8 and ferroptosis. The prediction from the JASPAR database indicated potential binding sites for HOXD8 within the promoters of GPX4 and SLC7A11 (Supporting Information S1: Figure S2A). Subsequent, Co‐IP assays revealed significant enrichment of GPX4 and SLC7A11 promoter by HOXD8, thereby confirming the binding of HOXD8 to the promoters of GPX4 and SLC7A11 (Supporting Information S1: Figure S2B). Furthermore, upon infecting CPT MSCs with lenti‐HOXD8 vector, we observed not only upregulation of HOXD8 expression but also enhanced expression of GPX4 and SLC7A11 (Supporting Information S1: Figure S2C). HOXD8 overexpression was also found to suppress the levels of Fe^2+^ and lipid ROS in CPT MSCs (Supporting Information S1: Figure S2D,E). Additionally, our previous study identified HOXD8 as a key factor in osteogenic differentiation of MSCs.^5^, ^10^ In this study, TRIM21 knockdown increased HOXD8 expression in CPT MSCs (Figure 2A). UbiBrowser prediction showed that the E3 ubiquitination ligase TRIM21 had binding sites to HOXD8 (Figure 2B) and that TRIM21 was reported to be involved in osteogenic differentiation.^15^ Here, we also found that both endogenous and exogenous TRIM21 and HOXD8 were immunoprecipitated from each other (Figure 2C,D). This further confirmed that TRIM21 interacted with HOXD8. The degradation of HOXD8 protein was delayed in CPT MSCs by knockdown of TRIM21, and the effect was further augmented by treatment with the proteasome inhibitor MG132 (Figure 2E). Meanwhile, TRIM21 knockdown increased the stability of HOXD8 protein in CPT MSCs under treatment with the protein synthesis inhibitor CHX (Figure 2F). Furthermore, exogenous overexpression of TRIM21 promoted ubiquitination and degradation of HOXD8 (Figure 2G). However, knockdown of TRIM21 suppressed HOXD8 ubiquitination and degradation in CPT MSCs (Figure 2H). The above results demonstrated that TRIM21 ubiquitinated and degraded HOXD8 in CPT MSCs.

### 3.3 Knockdown of ELAVL1 suppressed ferroptosis to promote osteogenic differentiation of CPT MSCs

**FIGURE 3:**
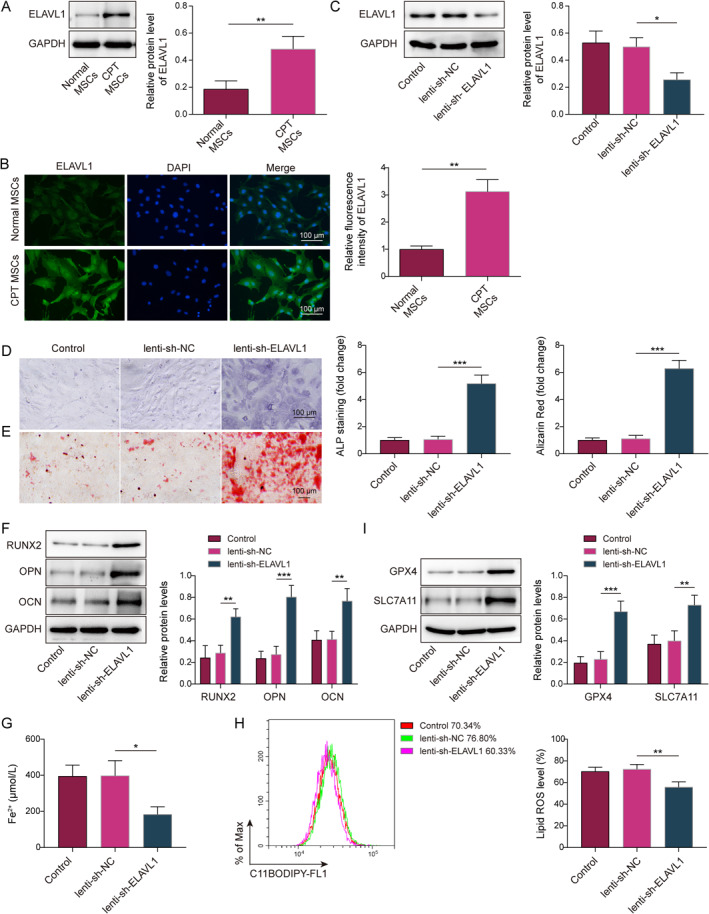
Knockdown of ELAVL1 suppressed ferroptosis to promote osteogenic differentiation of CPT mesenchymal stem cells (MSCs). (A) ELAVL1 expression in normal MSCs and CPT MSCs were assessed using the western blot. (B) Expression and localization of ELAVL1 were analyzed in normal MSCs and CPT MSCs using the IF staining (scale bar = 100 μm). CPT MSCs were infected with lenti‐sh‐ELAVL1 or lenti‐sh‐NC and induced osteogenic differentiation. (C) Western blot detection of ELAVL1 expression; (D) alkaline phosphatase (ALP) staining assay experiments using the ALP staining kit (scale bar = 100 μm); (E) cellular matrix mineralization was examined using the ARS staining kit (scale bar = 100 μm); (F) RUNX2, OPN, and OCN expression were assessed using the western blot. (G and H) Fe^2+^ content and lipid reactive oxygen species level in erastin‐treated MSCs were measured using the kit; (I) Western blot was applied to analyze ferroptosis‐related factors and GPX4 and SLC7A11 expression in erastin‐treated MSCs. *n* = 3. *p < 0.05, **p < 0.01, and ***p < 0.001.

ELAVL1 is thought to be a critical factor in promoting ferroptosis, and it can significantly inhibit osteogenic differentiation in MSCs.^22^, ^23^ In here, ELAVL1 expression was markedly elevated in CPT MSCs compared with normal periosteal‐derived MSCs (Figure 3A,B). Subsequently, CPT MSCs were infected with lenti‐sh‐ELAVL1 and induced osteogenic differentiation. In lenti‐sh‐ELAVL1‐infected CPT MSCs, ELAVL1 was downregulated, indicating that it was successfully knocked down (Figure 3C). Knockdown of ELAVL1 promoted osteogenic differentiation of CPT MSCs and upregulated RUNX2, OPN, and OCN expression (Figure 3D–F). After further induction of ferroptosis using erastin, knockdown of ELAVL1 resulted in a reduction of Fe^2+^ content and lipid ROS content in CPT MSCs, whereas the expression of GPX4 and SLC7A11 were elevated (Figure 3G–I and Supporting Information S1: Figure S1B). This showed that ELAVL1 was upregulated of periosteum‐derived MSCs in CPT, whereas knockdown of ELAVL1 inhibited ferroptosis and enhanced osteogenic differentiation of MSCs.

### 3.4 ELAVL1 increased the stability of TRIM21 mRNA

**FIGURE 4:**
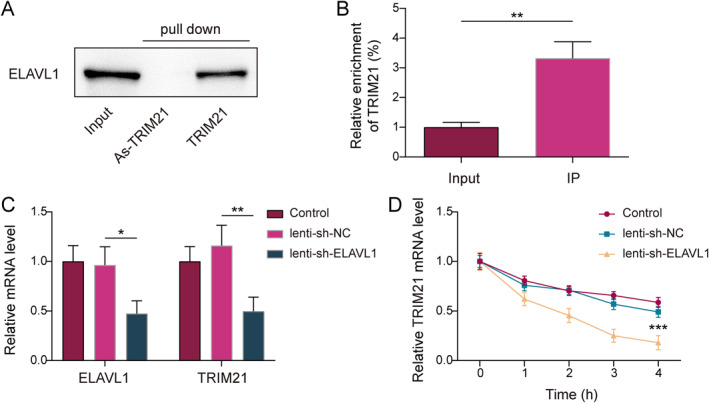
ELAVL1 increased the stability of TRIM21 mRNA. (A and B) RNA pulldown and RIP detection of ELAVL1 binding to TRIM21 mRNA in CPT mesenchymal stem cells (MSCs). (C) Levels of ELAVL1 and TRIM21 mRNA in ELAVL1‐knockdown CPT MSCs were measured using RT‐qPCR. (D) Lenti‐sh‐ELAVL1 or lenti‐sh‐NC was used to infect CPT MSCs. After infection, actinomycin D (5 μg/ml) was added for 0, 2, and 4 h. RT‐qPCR was used to determine levels of remaining TRIM21 mRNA. *n* = 3. *p < 0.05, **p < 0.01, and ***p < 0.001.

As shown in Figure 4A, the biotin‐labeled TRIM21 RNA probe pulled down the ELAVL1 protein. Meanwhile, TRIM21 mRNA was significantly enriched by immunoprecipitation antibody using anti‐ELAVL1 (Figure 4B). The above results demonstrated that ELAVL1 interacted with TRIM21 mRNA. As illustrated in Figure 4C, knockdown of ELAVL1 reduced ELAVL1 and TRIM21 mRNA levels. In addition, we found the binding between ELVALV1 and TRIM21 mRNA decreased due to ELAVL1 inhibition (Supporting Information S1: Figure S3A). Concurrently, we constructed luciferase reporter vectors containing the wild type (TRIM21‐wt) and mutant 3′UTR of TRIM21 (TRIM21‐Mut). In 293 T cells, transfection with sh‐ELAVL1 significantly reduced the luciferase activity of the TRIM21‐wt, whereas no significant change was observed in the mutant group (Supporting Information S1: Figure S3B,C). Meanwhile, knockdown of ELAVL1 reduced the level of TRIM21 mRNA in actinomycin D‐treated CPT MSCs (Figure 4D). Furthermore, to further elucidate the regulatory mechanism of ELAVL1 on TRIM21, we applied the inhibitors of PI3K (LY294002) and NF‐κB (BAY11‐7082), respectively, in CPT MSCs. Research has reported that NF‐κB can induce the upregulation of ELAVL through the PI3K/AKT signaling pathway.^24^ Our validation experiments revealed that as the ratio of p‐AKT to total AKT decreased as the concentration of LY294002 increased, and the expression of ELAVL1 was downregulated in a dose‐dependent manner (Supporting Information S1: Figure S3D). Similarly, NF‐κB inhibitor was also demonstrated to downregulate the expression of nuclear NF‐κB and ELAVL1 in a concentration‐dependent manner (Supporting Information S1: Figure S3E). The above results elucidated that ELAVL1 was activated by PI3K/AKT/NF‐κB axis and increased the stability of TRIM21 mRNA in CPT MSCs.

### 3.5 ELAVL1 promoted ferroptosis by regulating the TRIM21/HOXD8 axis to inhibit osteogenic differentiation of CPT MSCs

**FIGURE 5:**
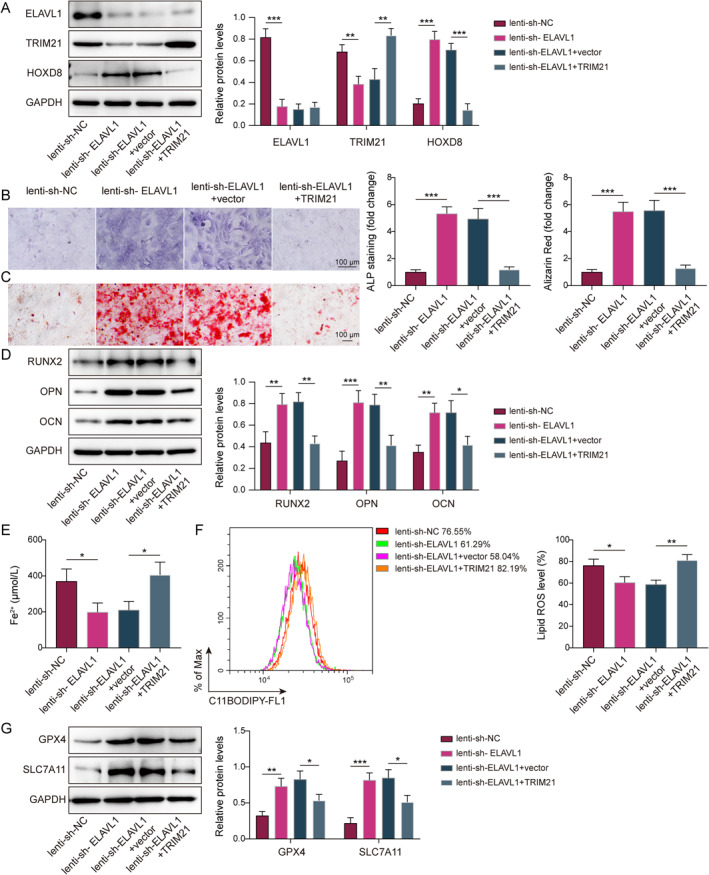
ELAVL1 promoted ferroptosis by regulating the TRIM21/HOXD8 axis to inhibit osteogenic differentiation of CPT mesenchymal stem cells (MSCs). Lenti‐sh‐ELAVL1 or/and lenti‐TRIM21 were utilized to infect CPT MSCs. (A) The expression of ELAVL1, TRIM21, and HOXD8 was assayed with the western blot method. (B) Alkaline phosphatase staining assays were performed using kits (scale bar = 100 μm). (C) ARS staining was used to examine mineralized nodules (scale bar = 100 μm). (D) Osteogenesis‐related marker genes RUNX2, OPN, and OCN expression were measured using the western blot. (E and F) Fe^2+^ content and lipid reactive oxygen species content of erastin‐treated CPT MSCs were assayed using the kit. (G) Western blot was applied to examine GPX4 and SLC7A11 expression in erastin‐treated CPT MSCs. *n* = 3. *p < 0.05, **p < 0.01, and ***p < 0.001.

Lenti‐sh‐ELAVL1 and lenti‐TRIM21 were utilized to knockdown ELAVL1 and overexpress TRIM21 in CPT MSCs. The lenti‐TRIM21 successfully upregulated TRIM21 levels in CPT MSCs (Supporting Information S1: Figure S3F,G). Compared with lenti‐sh‐NC, ELAVL1 knockdown decreased ELAVL1 and TRIM21 expression, whereas HOXD8 expression was increased. In contrast, TRIM21 overexpression upregulated TRIM21 expression and downregulated HOXD8 levels (Figure 5A). Moreover, TRIM21 overexpression eliminated the promotional effect of ELAVL1 knockdown on osteogenic differentiation of CPT MSCs and expression of osteogenesis‐related marker (Figure 5B–D). Furthermore, the lipid ROS production in CPT MSCs was suppressed by ELAVL1 inhibition, but meanwhile, TRIM21 overexpression partly reversed the inhibitory effect of ELVAVL1 knockdown (Supporting Information S1: Figure S1C). In erastin‐treated CPT MSCs, knockdown of ELAVL1 reduced Fe^2+^ content and lipid ROS content and increased GPX4 and SLC7A11 expression, whereas overexpression of TRIM21 reversed these changes (Figure 5E–G). Therefore, ELAVL1 suppressed CPT osteogenic differentiation of MSCs by modulating TRIM21/HOXD8 axis, thereby triggering ferroptosis.

## 4 DISCUSSIONS

CPT is a rare and extremely difficult disease to treat, and its etiology and pathogenesis remain unclear.^25^ Osteogenic differentiation of periosteum‐derived MSCs is a major progression of CPT bone regeneration and healing.^10^ Activation of ferroptosis could inhibit osteogenic differentiation of MSCs.^8^ In addition, iron overload or iron deficiency is strongly associated with bone homeostasis.^26^ However, there are no studies related to ferroptosis and CPT. Our study elucidated that ELAVL1 enhanced the stability of TRIM21 mRNA, thereby promoting degradation of HOXD8, which triggered ferroptosis and ultimately inhibited osteogenic differentiation of CPT MSCs.

Our previous study exhibited that HOXD8 expression was clearly downregulated in CPT MSCs.^5^, ^10^ However, the specific reasons for its downward adjustment deserve further exploration. Ubiquitination modification is an important protein degradation mechanism that is widespread in eukaryotes.^13^ TRIM21 is an E3 ubiquitination ligase that can participate in bone‐related pathophysiological processes by ubiquitinating key proteins. A study revealed that TRIM21 induced disc degeneration by mediating ubiquitination of hypoxia‐inducible factor 1 subunit *α*.^27^ Another study demonstrated that TRIM21 promoted ubiquitination and degradation of Akt to inhibit osteogenic differentiation of MSCs.^15^ It also found that TRIM21 expression was reduced during MSCs osteogenesis, and it suppressed osteogenic differentiation of MSCs.^15^ Liu et al.^28^ discovered that TRIM21 deficiency promoted bone formation, which highlights its critical role in bone formation. Our study showed for the first time that TRIM21 ubiquitinated and degraded HOXD8. In addition, TRIM21 expression was upregulated of periosteum‐derived MSCs in CPT. Meanwhile, knockdown of TRIM21 promoted osteogenic differentiation of CPT MSCs. More and more research showed that TRIM21 promoted ferroptosis. Recent studies identified that TRIM21 promoted ferroptosis through the SQSTM1‐NRF2‐KEAP1 axis to increase titers of H5N1 highly pathogenic avian influenza virus.^29^ Hou et al.^30^ demonstrated that silencing of TRIM21 inhibited ferroptosis to attenuate cardiotoxicity. The literature also reported that TRIM21 promoted ferroptosis by ubiquitinating degradation‐associated proteins.^17^ In our study, knockdown of TRIM21 also inhibited ferroptosis in periosteal‐derived MSCs in CPT. Overall, our study illustrated that TRIM21 ubiquitinated and degraded HOXD8 and promoted ferroptosis to inhibit osteogenic differentiation of CPT MSCs.

ELAVL1, as an RBP, was reported to inhibit osteogenic differentiation of bone‐marrow‐derived MSCs via regulating the stability of extracellular matrix‐related genes.^19^ Li et al.^3^ found that ELAVL1 mediated the stability of HOX transcript antisense RNA to inhibit osteogenic differentiation of MSCs and bone formation. In addition, an in vivo study indicated that ELAVL1 expression was increased in bone tissue of diabetic mice.^22^ Our research also clarified that ELAVL1 expression was upregulated in MSCs of CPT periosteal origin. Moreover, ELAVL1 increased the stability of TRIM21 mRNA. Knockdown of ELAVL1 decreased the level of TRIM21 to promote osteogenic differentiation of periosteum‐derived MSCs in CPT. In addition, numerous publications suggested that ELAVL1 could promote cellular ferroptosis in a variety of physiological pathways.^20^, ^31^, ^32^ For example, ELAVL1 was activated by FOXC1 to promote cardiomyocyte ferroptosis during myocardial ischemia‐reperfusion injury.^31^ Silencing of ELAVL1 suppressed neuronal ferroptosis in cerebral ischemia‐reperfusion rats by mediating DNMT3B mRNA stability.^20^ Importantly, Ren et al.^22^ revealed that knockdown of ELAVL1 promotes bone formation by inhibiting iron overload in bone tissue of diabetic mice. In this work, we also demonstrated that knockdown of ELAVL1 inhibited ferroptosis of periosteum‐derived MSCs in CPT. Moreover, the above effects were reversed by overexpression of TRIM21. Thus, the present study evidenced that ELAVL1 promoted ferroptosis by regulating the TRIM21/HOXD8 axis, which in turn inhibited osteogenic differentiation periosteum‐derived MSCs in CPT.

In conclusion, our study revealed a novel mechanism whereby ELAVL1 bound to TRIM21 for promoting ubiquitination and degradation of HOXD8, which triggered ferroptosis and consequently inhibited osteogenic differentiation of CPT MSCs. Conversely, the present research is not without its limitations. Notably, clinical and in vivo experiments have not been incorporated into the current study framework. Moving forward, we intend to augment our research through the integration of multilevel experimental approaches. This will enable a more comprehensive elucidation and validation of the underlying mechanisms. Our findings illuminated for the first time the critical effect of ferroptosis in CPT development. It offered new theories for the etiology and clinical treatment of CPT. However, this molecular mechanism needs to be further validated at the animal level.

## AUTHOR CONTRIBUTIONS

Weihua Ye and Guanghui Zhu designed this study. Zheng Liu, Yaoxi Liu, Han Xiao, Qian Tan, and An Yan collected the materials and performed the experiments. Weihua Ye analyzed the data and wrote the manuscript. Guanghui Zhu revised the manuscript. All authors have read and approved the final version of the manuscript.

## CONFLICT OF INTEREST STATEMENT

The authors declare no conflicts of interest.

## ETHICS STATEMENT

This study involved the use of signed informed consent forms for all participants, and the work was overseen by the Ethics Committee of Hunan Children's Hospital.

## Supporting information

Supporting Information S1

## Data Availability

All data generated or analyzed during this study are included in this published article.
