# Supplementary material for: ELAVL1 promotes ferroptosis via the TRIM21/HOXD8 axis to inhibit osteogenic differentiation in congenital pseudoarticular tibia‐derived mesenchymal stem cells
Source: J Cell Commun Signal. 2025 May 21;19(2):e70016. doi: 10.1002/ccs3.70016 (PMC12094168; doi:10.1002/ccs3.70016)
Supplement: Supplementary file 1 — Supporting Information S1 [file CCS3-19-e70016-s001.pdf]

Supplementary Materials

Supplementary Figure and Figure legends

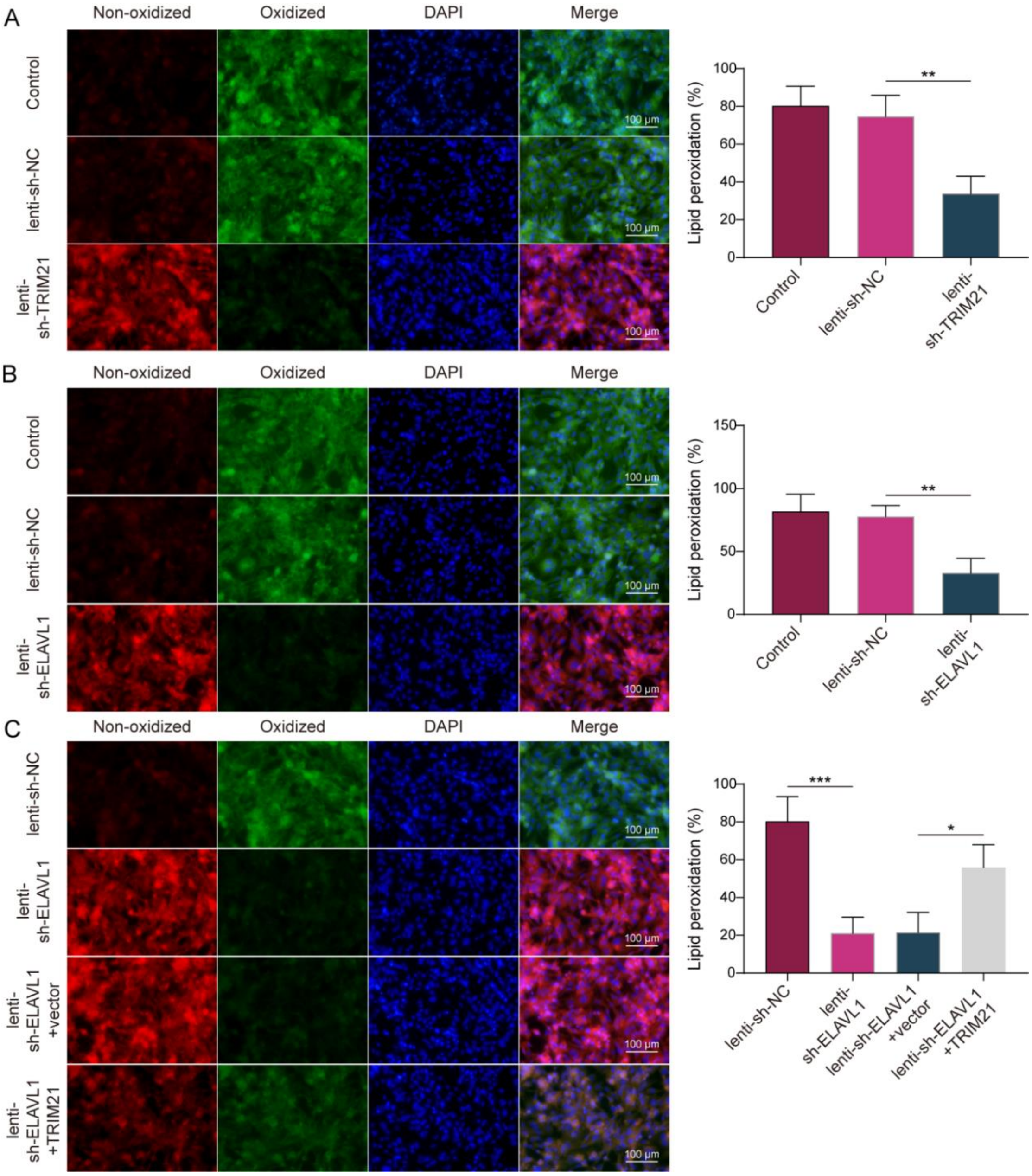

Fig. S1. Lipid ROS was evaluated in CPT MSCs. (A) CPT MSCs were infected with lenti-sh-TRIM21 or lenti-sh-NC, and lipid ROS production was detected by C11-BODIPY and imaged by fluorescence microscope. Red, reduced dye; green, oxidized dye. Scale bar = 100 μm; (B) CPT MSCs were infected with lenti-sh-ELAVL1 or lenti-sh-NC, and lipid ROS production was detected by C11-BODIPY and imaged by

fluorescence microscope. Red, reduced dye; green, oxidized dye. Scale bar = 100 μm; (C) CPT MSCs were infected with lenti-sh-ELAVL1 and/or lenti-TRIM21, and lipid ROS production was detected by C11-BODIPY and imaged by fluorescence microscope. Red, reduced dye; green, oxidized dye. Scale bar = 100 μm. n=3. *P<0.05, **P<0.01 and ***P<0.001.

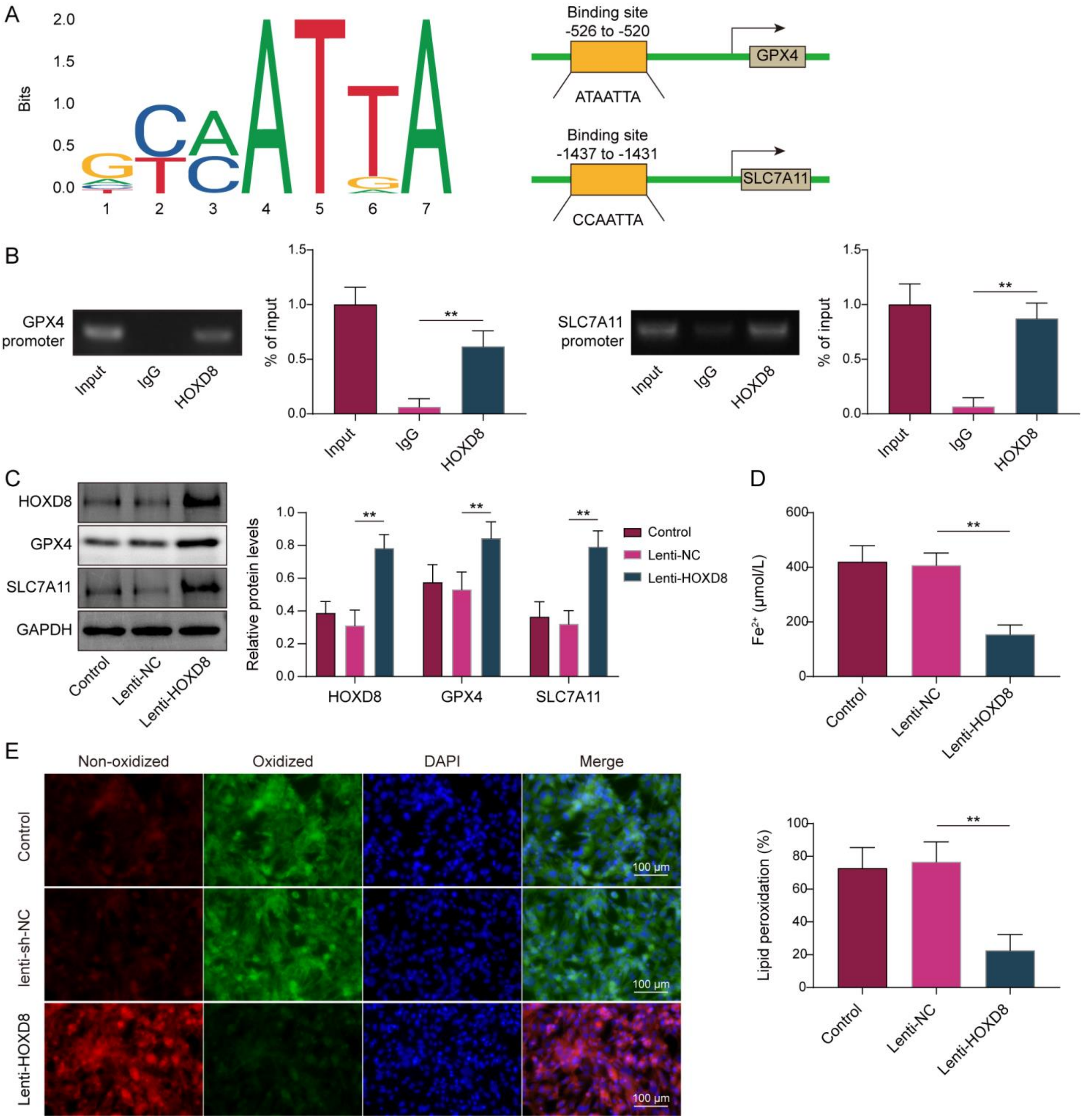

Fig. S2. HOXD8 repressed ferroptosis in CPT MSCs. (A) JASPAR database was

used to predict the binding of HOXD8 to GPX4 and SLC7A11 promoter. (B) ChIP assays were performed to detect of HOXD8 binding to GPX4 and SLC7A11 promoter in CPT MSCs. CPT MSCs were infected with lenti-HOXD8 or lenti-NC. (C) Levels of HOXD8, GPX4 and SLC7A11 protein in HOXD8-overexpression CPT MSCs were measured using Western blot; (D) Fe^{2+} content in MSCs were measured by the kit. (E) Lipid ROS production was detected by C11-BODIPY and imaged by fluorescence microscope. Red, reduced dye; green, oxidized dye. Scale bar = 100 μm n=3. ***P*<0.01.

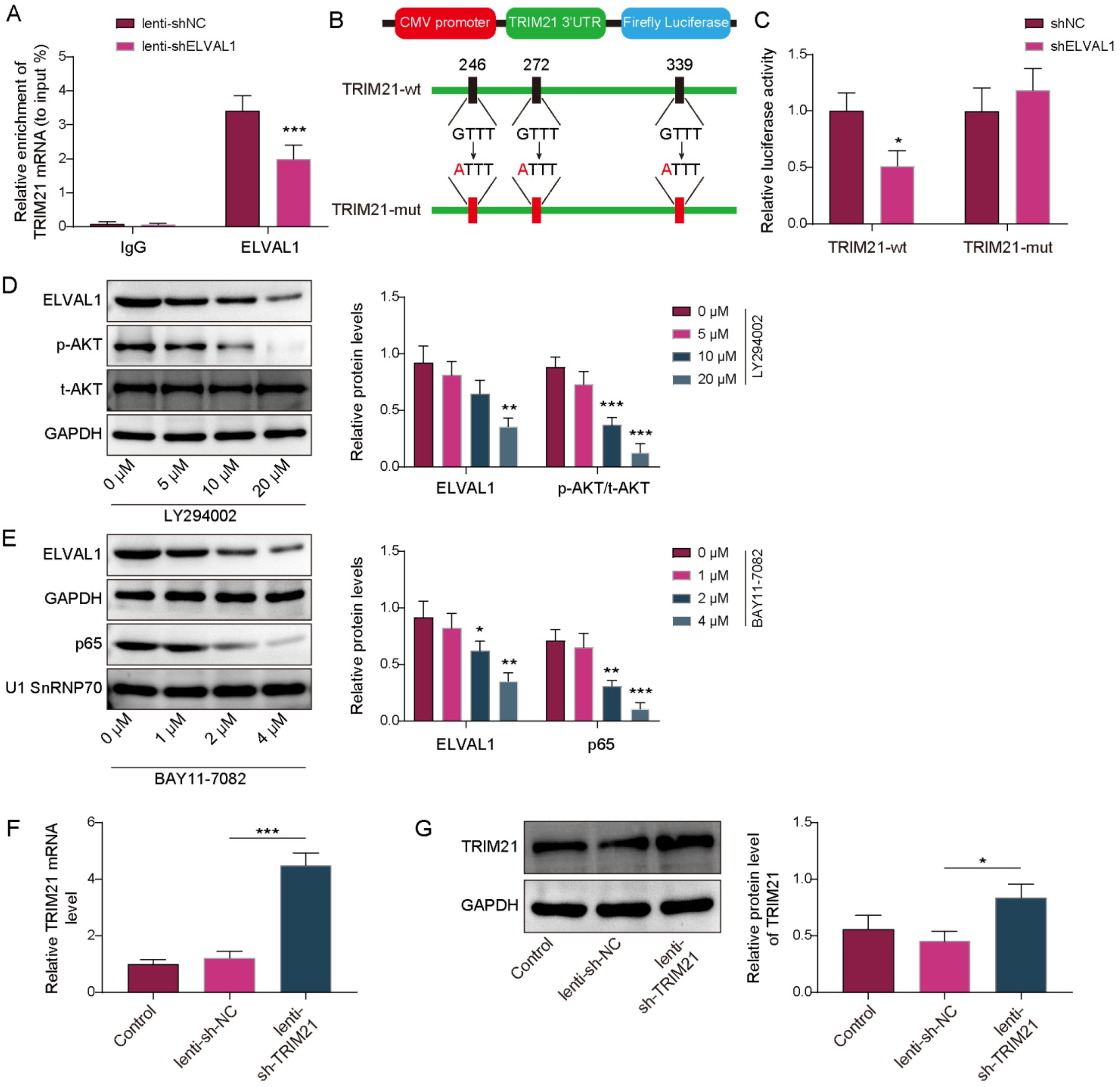

Fig. S3. ELAVL1 bound to TRIM21 mRNA, and PI3K/AKT induced p65 nuclear entry enhanced ELAVAL1 levels. Lenti-sh-ELAVL1 or lenti-sh-NC was used to infect CPT MSCs. (A) RIP detection of ELAVL1 binding to TRIM21 mRNA in CPT MSCs. (B) Schematic diagram of TRIM21-wt and TRIM21-Mut constructs. (C) Dual-luciferase assay was used to detect ELAVL1 binding to TRIM21 mRNA in 293T cells. (E) The protein levels of ELAVL1, p-AKT and t-AKT in CPT MSCs treated with PI3K inhibitor LY294002 (0, 5, 10, 20 μM) were evaluated by Western blot. (E) The protein levels of ELAVL1, and nuclear p65 in CPT MSCs treated with NF-κB inhibitor BAY11-7082 (0, 1, 2, 4 μM) were evaluated by Western blot. (F-G) Lenti-sh-TRIM21 or lenti-sh-NC was used to infect CPT MSCs, and TRIM21 expression was tested by RT-qPCR(F) and Western blot(G). n=3. *P<0.05, **P<0.01 and ***P<0.001.
